# Qishen Yiqi Dripping Pill Protects Diabetic Nephropathy by Inhibiting the PI3K-AKT Signaling Pathways in Rats

**DOI:** 10.1155/2022/6239829

**Published:** 2022-06-17

**Authors:** Shaohua Li, Guangbiao Xu

**Affiliations:** ^1^Department of Nephrology, The First Affiliated Hospital (Southwest Hospital) of Army Medical University, Chongqing 400038, China; ^2^Department of Nephrology, Taizhou Hospital of Zhejiang Province, Taizhou 317000, China

## Abstract

This study aimed to explore the potential mechanisms of Qishen Yiqi dripping pill (QYDP) against diabetic nephropathy (DN) through network pharmacology and animal experiments. The components and targets of QYDP and DN-related targets were retrieved from public databases. A total of 116 compounds and 160 targets of QYDP anti-DN were obtained. The top 10 hub targets including AKT1, TNF, ALB, INS, PPARG, IL-6, IL-1B, VEGF-A, JUN, and MAPK3 were screened by Cytoscape software. Then, the key targets of QYDP were enriched in 1815 Gene Ontology (GO) entries (*P* < 0.01) and 159 Kyoto Encyclopedia of Genomes and Genomes (KEGG) pathways (*P* < 0.01), mainly including the AGE-RAGE signaling pathway in diabetic complications and the PI3K-AKT signaling pathway. In animal experiments, the results of an ELISA assay showed that QYDP could regulate the expression levels of kidney function-related indexes and reduce the expression of inflammatory factors. qRT-PCR and western blot results showed that QYDP regulated the expression of hub genes. In conclusion, this study shows that QYDP could treat DN by antioxidative and antiinflammatory activity and inhibiting the PI3K-AKT signaling pathway.

## 1. Introduction

Diabetic nephropathy (DN) is the end-stage renal disease of type 2 diabetes mellitus (T2DM) [1]. In 2021, 10.5% of the world's population will have diabetes, and this is on the rise [2]. Glomerular hyperfiltration and proteinuria are early clinical manifestations of DN [3]. The molecular pathogenesis of DN is poorly understood, and no new drugs have been approved for the treatment of DN in the past 20 years [4]. Therefore, there is an urgent need to find effective drug candidates for the treatment of DN.

Qishen Yiqi dropping pill (QYDP) is a traditional Chinese medicine (TCM) consisting of Radix Astragali (*Astragalus penduliflorus* Lam.), redroot sage (*Salvia miltiorrhiza* Bunge), pseudoginseng (*Panax pseudoginseng* Wall.), and fragrant rosewood (*Dalbergia odorifera* T.C. Chen). Studies have shown that the urinary serum excretion rate of diabetic patients is reduced by QYDP administration [5]. Moreover, QYDP intervention alleviates renal interstitial fibrosis in chronic kidney disease model rats with unilateral ureteral obstruction [6]. However, the action mechanisms of QYDP anti-DN is still unclear.

In this study, the network pharmacological method of TCM was used to predict the target and mechanism of QYDP in DN, and the results were verified by animal experiments. This study lays the foundation for a follow-up study of QYDP against DN.

## 2. Methods

### 2.1. QYDP Ingredient Collection and Target Prediction

Active ingredients of QYDP were extracted from the traditional Chinese medicine systems pharmacology database and analysis platform (TCMSP, https://tcmsp-e.com/) [7], Traditional Chinese Medicine Integrated Database (TCM-ID, https://www.megabionet.org/tcmid/) [8], and Herb Ingredient' Targets (HIT, https://lifecenter.sgst.cn/hit/) [9]. We collected active ingredients by setting the drug-likeness index so that the quantitative estimate of DL (QED) was ≥0.2.

The potential targets of QYDP were predicted by TCM-ID, TCMSP, HIT, and Search Tool for Interacting Chemicals (STITCH, https://stitch.embl.de) [10] databases and collected with the compound-target related score was greater than 400 in the STITCH database. Moreover, active ingredients and potential targets of QYDP were further screened by the binomial statistical mode [11].

### 2.2. Building DN Target Database

DN-related targets were derived from three databases: Online Mendelian Inheritance in Man (OMIM, https://omim.org) [12], DisGeNET (https://www.disgenet.org) [13], and GeneCard (https://www.genecards.org/) [14]. For filtering the targets, “diabetic nephropathy” was defined as a keyword, and the species was restricted to *Homo sapiens*, and targets with a correlation score above the median (≥1.81388) were selected in the GeneCard database. The results of the above 3 databases are merged and duplicate targets are removed.

### 2.3. Protein-Protein Interaction (PPI) Analysis

Using the STRING database [15], the potential targets of QYDP anti-DN were reanalyzed to obtain the PPI networks. The interaction files were downloaded from the STRING database and imported into Cytoscape software (version 3.8.2) to visualize the PPI networks and analyze the topology parameters.

### 2.4. Function Analysis

The Database for Annotation, Visualization, and Integrated Discovery (DAVID database, https://david.ncifcrf.gov) were used for Gene Ontology (GO) and Kyoto Encyclopedia of Genes and Genomes (KEGG) enrichment analysis of the potential targets. GO enrichment analysis includes biological processes (BP), cellular components (CC), and molecular functions (MF). The enrichment results of GO and KEGG were obtained by setting *P* < 0.01.

### 2.5. DN Mouse Model Construction and Treatment

The animal experiments were approved by the Ethics Committee of Taizhou Hospital of Zhejiang Province. Eight-week-old male C57BL/6J mice were obtained from Beijing HFK Bioscience Co., Ltd. (China), housed in a controlled room with a 12-hour light/dark cycle at 23 ± 2°C, and received food and water *ad libitum*. After one week of adaption, the DN mice model was established by intraperitoneal injection of 50 mg/kg streptozotocin (STZ, Sigma-Aldrich, MO, USA) for 5 days. Mice with blood glucose concentrations of 11–18 mM were considered successful mouse models of DN. Then, all mice were randomly divided into six groups (*n* = 6 per group): control group, STZ-induced DN model group, QYDP administered groups (low dose: QYDP 6 mg/ml gavage; middle-dose: QYDP 12 mg/ml gavage; high-dose: QYDP 24 mg/ml gavage); and a high-dose QYDP + 740Y-P (PI3K agonist, Sigma-Aldrich) group (QYDP 24 mg/ml gavage + 740Y-P 5 *μ*mol/kg intraperitoneal injection). After 5 weeks of administration, all mice were anesthetized with sodium pentobarbital (50 mg/kg, intraperitoneal injection) and then sacrificed by cervical dislocation. The blood and kidney samples were obtained for further analysis.

### 2.6. Measurement of Physiological Characteristics

After fasting for 6 hours in the morning, the body weight and fasting blood glucose (FBG) levels were measured once a week using a blood glucometer (Johnson and Johnson Milpitas, CA, USA). In addition, at the end of the experimental period, 24-hour urine volumes were collected from all mice in the metabolic cages.

### 2.7. ELISA Assay

ELISA kits (Beyotime, Shanghai, China) were used to measure the levels of albumin (ALB), blood urea nitrogen (BUN), serum creatinine (Scr), total cholesterol (TC), and triglyceride (TG) in the urine or serum of all mice according to the protocol of the manufacturer. Similarly, the levels of inflammatory cytokines (interleukin 6 (IL-6), IL-1*β*, and tumor necrosis factor-*α* (TNF-*α*)) in the serum of all mice were also measured by ELISA kits.

### 2.8. Hematoxylin and Eosin (HE) Staining

HE staining was performed according to the protocols previously reported [16]. Firstly, kidney tissues were fixed in 10% formalin buffer, embedded in paraffin, and cut into slices (thickness of 5 *μ*m). Next, the slices were stained with hematoxylin and eosin for 3 min using a light microscope (Olympus, Japan) to observe the histological images.

### 2.9. Quantitative Real-Time PCR (qRT-PCR) Assay

Total RNA was extracted from kidney tissues using TRIzol reagent (Invitrogen, CA, USA) and then reverse-transcribed into cDNA using PrimeScript RT Master Mix (TaKaRa, Japan) according to the manufacturer's instructions. PCR was performed using an SYBR Green kit (Agilent Technologies, TX, USA), and the reaction volume was 20 *μ*L per well, including 10 *μ*L of SYBR Green Mix, 1 *μ*L of cDNA, 2 *μ*L of primer pair mix, and 7 *μ*L of DNAse/RNAse-free H_2_O. The cycling conditions for qRT-PCR are as follows: initial denaturation at 95°C for 3 min, followed by 40 cycles of 95°C for 12 sec and 62°C for 40 s. Gene expression was assessed by the 2^−ΔΔCT^ method and GAPDH as the house gene. The primers are listed in Supplementary Table 1.

### 2.10. Western Blot

Proteins were extracted from kidney tissues using RIPA lysis buffer (Beyotime), loaded into a sodium dodecyl sulfate-polyacrylamide gel electrophoresis gel, and transferred into polyvinylidene fluoride membrane. After blocking in the 5% skim milk, membranes were incubated with primary antibodies against PPARG (1 : 1000, ^#^AP20705a, abcepta, China), VEGF-A (1 : 1000, ^#^AF5131, Affinity, China), PI3K (1 : 1000, ^#^4257, Cell Signaling Technology (CST), MA, USA), p-PI3K (1 : 1000, ^#^4228, CST), AKT (1 : 1000, ^#^9272, CST), and p-AKT (1 : 1000, ^#^9271, CST) overnight at 4°C, followed by incubated with secondary antibodies (1 : 2000, Abcam, UK) for 1 h. The bands were detected in an enhanced chemiluminescence detection system (Millipore, UK).

### 2.11. Statistical Analysis

Data were analyzed using Prism 8 (GraphPad, CA, USA) and expressed as mean ± standard deviation. One-way ANOVA followed by Tukey's tests was used to compare multiple groups. *P* < 0.05 was considered a statistically significant difference.

## 3. Results

### 3.1. QYDP Ingredient Collection and Target Prediction

A total of 170 active ingredients were obtained from TCMSP, TCM-ID, and HIT databases. Then, 152 active ingredients were further screened by QED ≥0.2. A total of 6,828 DN-related targets were collected from TCMSP, TCM-ID, HIT, and STITCH databases. Then, through the binomial statistical mode algorithm, we obtained 578 potential targets and 133 active ingredients.

### 3.2. Collection of DN-Related Targets

A total of 312, 109, and 1,189 DN-related targets were extracted from GeneCard, OMIM, and DisGeNET databases, respectively. And, 1,333 DN-related targets remained followed by deleting the duplicate values.

### 3.3. Construction of the Herb-Compound-Target (H-C-T) Network

**Figure 1:**
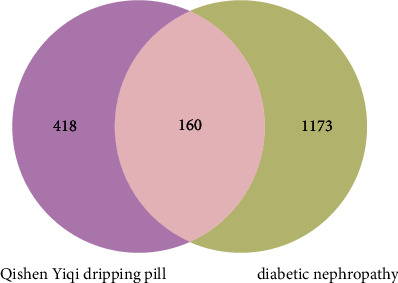
Venn diagram of Qishen Yiqi dripping pill (QYDP) related targets and diabetic nephropathy (DN) related targets.

**Figure 2:**
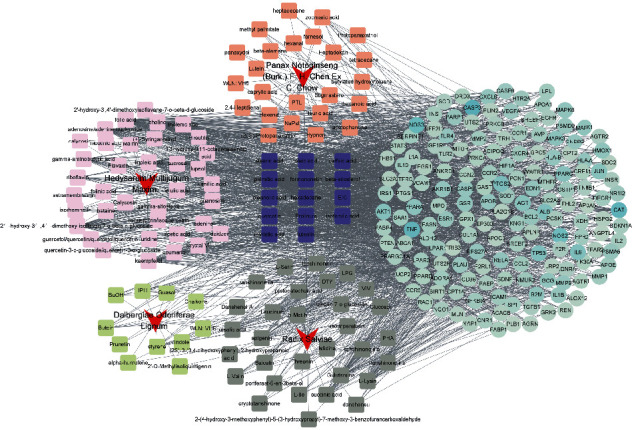
The herb-compound-target (H-C-T) network. The circle is the key target of QYDP acting on DN, the square represents the compound contained in QYDP, the blue square represents the compound contained in various herbs, and the red dart shape represents different herbs.

As shown in Figure 1, using Venny (version 2.1) software, we have redrived 160 key targets of QYDP against DN and then mapped 116 active compounds of QYDP against DN. The H-C-T network of QYDP was visualized by Cytoscape (Figure 2). This network contained 281 nodes and 1850 edges. More importantly, by further analyzing the degree of each node, we have found that palmitic acid, folic acid, quercetin, choline, and oleic acid could potentially be the most important active compounds of QYDP.

### 3.4. PPI Network Construction and Hub Targets Network Identification

**Figure 3:**
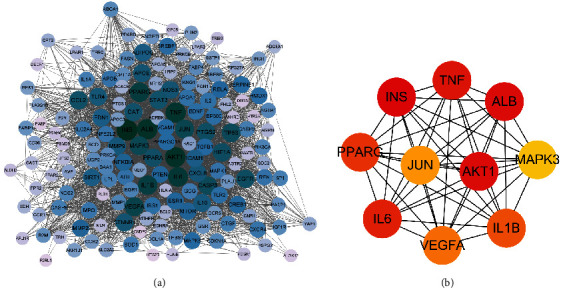
(a) The protein-protein interaction (PPI) network of 160 key targets was constructed with the STRING database. (b) The top 10 central genes for QYDP anti-DN were obtained using the degree algorithm. Node size and color are positively correlated with the degree value.

The STRING database was used to acquire PPI relationships of 160 key targets of QYDP anti-DN. The PPI network constructed by Cytoscape and consisted of 160 nodes and 3362 edges (Figure 3(a)). The average node degree of this network was 42. In addition, the top 10 targets with the highest node degrees were screened and a hub gene network constructed using Cytohubba, a plug-in of Cytoscape. The hub gene network consisted of AKT1, TNF, ALB, INS, PPARG, IL-6, IL-1B, VEGF-A, JUN, and MAPK3, and contained 10 nodes and 45 edges (Figure 3(b)).

### 3.5. GO and KEGG Enrichment Analysis of Key Targets

**Figure 4:**
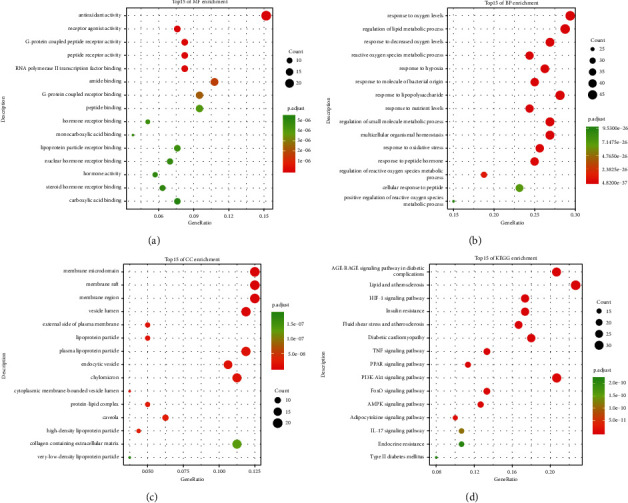
Gene Ontology (GO) and Kyoto Encyclopedia of Genomes and Genomes (KEGG) enrichment analysis. (a) Molecular function (MF). (b) Biological process (BP). (c) Cellular component (CC). (d) KEGG.

To further explore the biological functions of the 160 key targets, GO enrichment analysis was performed on the DAVID database and obtained 84 terms of MF, 1679 terms of BP, and 52 terms of CC (*P* < 0.01). As shown in Figure 4(a)–4(c), we exhibited the top 15 most enriched MF, BP, and CC terms. In these bubble charts, we found that QYDP administered DN may be associated with antioxidant activity, involved in response to oxygen levels and regulation of lipid metabolic processes, and located in the membrane microdomain, membrane raft, and membrane region.

**Figure 5:**
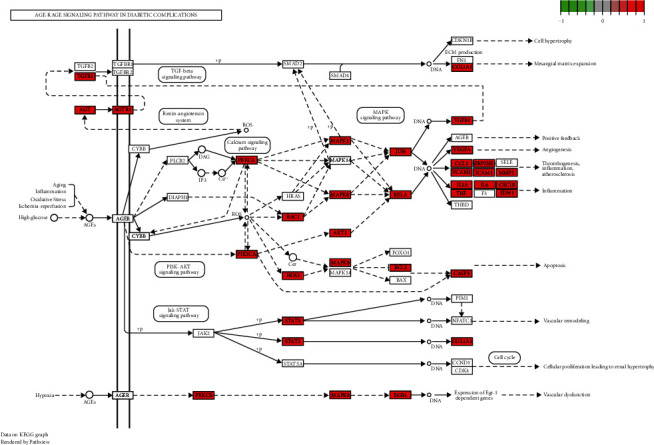
The map of the AGE-RAGE signaling pathway in diabetic complications. Red boxes represent key targets in the pathway.

Furthermore, we have harvested 159 terms of KEGG enrichment according to *P* < 0.01 and the top 15 pathways are displayed in Figure 4(d). The pathways result was intensively enriched in the AGE-RAGE signaling pathway in diabetic complications (Figure 5), lipid and atherosclerosis, PI3K-AKT signaling pathway, etc. Interestingly, the signaling transduction of the AGE-RAGE signaling pathway in diabetic complications was mainly composed of genes from TGF-*β*, MAPK, PI3K-AKT, and JAK-STAT pathways, further suggesting the importance of these pathways in the pathogenesis of DN (Figure 5). These results suggest that the therapeutic effects of QYDP in DN may be partly through its immunomodulatory effects.

**Figure 6:**
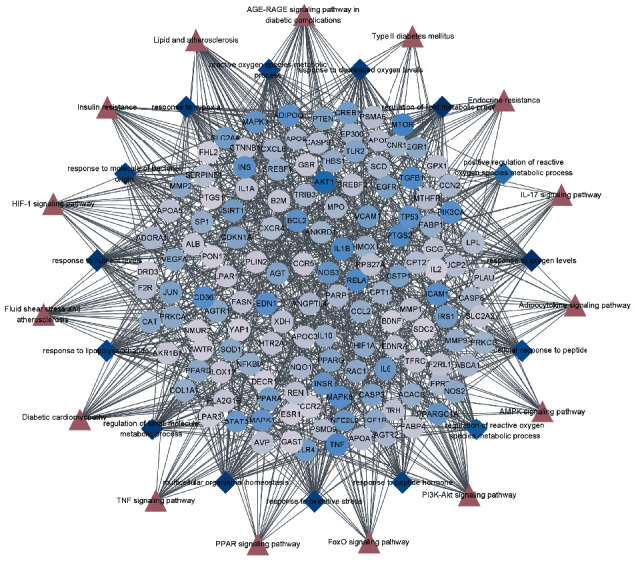
The target-BP-pathway (T-BP-P) network. The circles represent the key targets of QYDP acting on DN, the diamonds represent the top 15 BP enrichment analysis, and the triangles represent the top 15 signaling pathways.

The target-BP-pathway (T-BP-P) network showed the associations of the key targets with the top 15 BP and top 15 pathways (Figure 6). The network consisted of 169 nodes and 934 edges. This result revealed that the therapeutic effects of QYDP on DN may be achieved by participating in multiple biological processes and regulating multiple signaling pathways.

### 3.6. Effect of QYDP on the Physiological and Serological Changes in DN Mice

**Figure 7:**
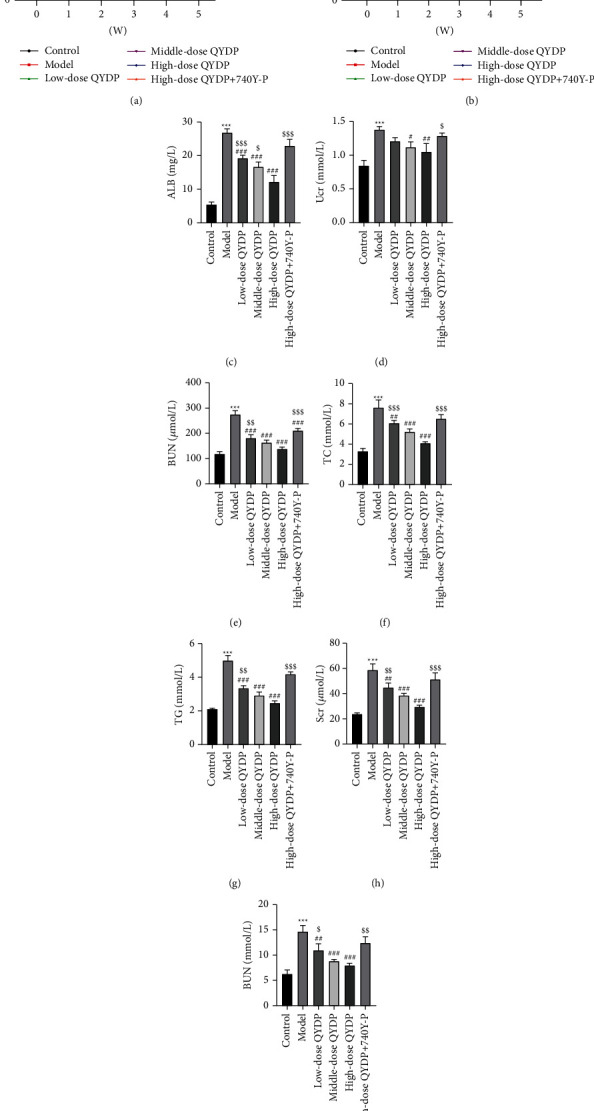
Effect of QYDP on the physiological and serological changes in DN mice. (a) Body weight. (b) Fasting blood glucose (FBG). (c–e). The levels of albumin (ALB), blood urea nitrogen (BUN), and creatinine (cr) in the urine of all mice. (f–i). The levels of total cholesterol (TC), triglyceride (TG), serum creatinine (Scr), and blood urea nitrogen (BUN) in the serum of all mice.  ^*∗*^ ^*∗*^ ^*∗*^*P* < 0.001 vs. control group; ^#^*P* < 0.05, ^##^*P* < 0.01, and ^###^*P* < 0.001 vs. model group; ^$^*P* < 0.05, ^$$^*P* < 0.01, and ^$$$^*P* < 0.001 vs. high-dose QYDP group.

To investigate the effect of QYDP on the physiological and serological changes in DN mice, we measured physiological and serological indicators including body weight, FBG, ALB, Ucr, BUN, TC, TG, and Scr. As shown in Figures 7(a) and 7(b), compared with the control group, the body weight and FBG have a significant difference in the model group. QYDP administration in DN mice markedly increased the body weight and decreased the FBG. Moreover, high-dose QYDP has a better therapeutic effect. In addition, to verify the role of the PI3K-AKT signaling pathway in QYDP treatment of DN, high-dose QYDP combined with a PI3K-AKT pathway agonist (740Y-P) was administered to DN mice. As expected, the addition of 740Y-P significantly reversed the improved effects of high-dose QYDP on body weight and fasting blood glucose in DN mice.

For the urine examination of DN mice, we detected the changes in ALB, Ucr, and BUN. As shown in Figures 7(c)–7(e), ALB, Ucr, and BUN were significantly increased in the urine of DN mice compared with controls. The addition of low, middle, and high doses of QYDP can significantly improve this phenomenon. However, the addition of 740Y-P weakened the effect of QYDP. Furthermore, the related indicators (TC, TG, Scr, and BUN) of blood detection (Figures 7(f)–7(i)) in DN mice showed the same trend as the indicators related to urine detection.

### 3.7. Effects of QYDP on Histopathological Changes and Inflammatory Response of Kidney in DN Mice

**Figure 8:**
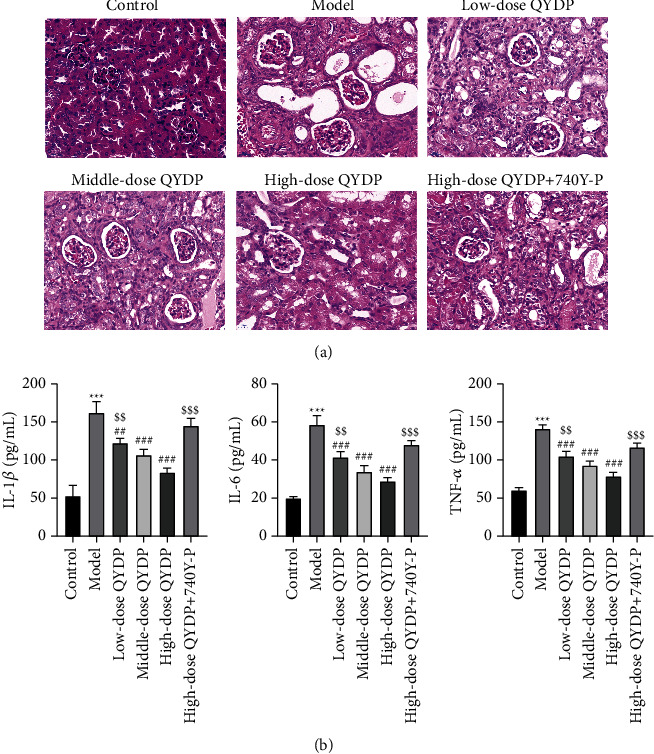
Effects of QYDP on histopathological changes and inflammatory response of the kidney in DN mice. (a) Histopathological changes in the kidneys were observed by HE staining. (b) ELISA to detect the expression of inflammatory factors (interleukin 6 (IL-6), IL-1*β*, and tumor necrosis factor-*α* (TNF-*α*)).  ^*∗*^ ^*∗*^ ^*∗*^*P* < 0.001 vs. control group; ^##^*P* < 0.01 and ###*P* < 0.001 vs. model group; ^$$^*P* < 0.01 and ^$$$^*P* < 0.001 vs. high-dose QYDP group.

Next, we observed histopathologic changes in kidney tissues using HE staining (Figure 8(a)). The results of HE staining showed that the size and shape of the kidney tissue of the mice in the control group were normal, and the structure was intact. The renal tissue of the mice in the model group was severely damaged, with increased glomerular volume, thickened glomerular basement membrane, vacuolar degeneration of renal tubules, and infiltration of inflammatory cells with fibrosis. After treatment with different doses of QYDP, the volume of glomeruli in each group decreased, the thickening of the glomerular basement membrane, the vacuolar degeneration of renal tubules, and the infiltration of inflammatory cells were improved to varying degrees. Compared with the QYDP treatment group, the high-dose QYDP + 740Y-P group showed thinner renal cortex, unclear cortical and medulla boundaries, diffused proliferation of mesangial cells and stroma, stenosis of some capillary lumen, focal atrophy of renal tubules, and interstitial atrophy, inflammatory cell infiltration. But still not as serious as the model group.

Additionally, to determine the inhibitory effect of QYDP on inflammation in DN mice, we measured the expression of inflammatory cytokines in serum, including IL-1*β*, IL-6, and TNF-*α*. Compared with the control group mice, the expressions of IL-6, IL-1*β*, and TNF-*α* in the serum of the model group mice were increased. In addition, the intervention of QYDP attenuated the renal inflammatory response and the secretion of these cytokines was significantly reduced, while the addition of 740Y-P attenuated the antiinflammatory effect of QYDP (Figure 8(b)).

### 3.8. Effects of QYDP on the Expression of Hub Genes and PI3K-AKT Pathway Genes in DN Mice

**Figure 9:**
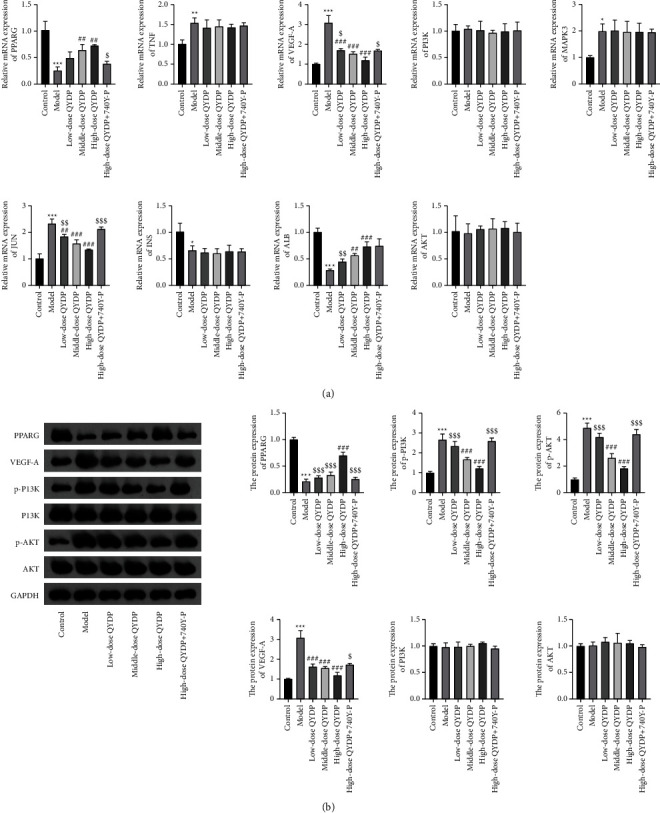
Effects of QYDP on the expression of hub genes and PI3K-AKT pathway genes in DN mice. (a) The relative expression of PPARG, TNF, VEGF-A, PI3K, MAPK3, JUN, INS, ALB, and AKT were detected by qRT-PCR. (b) The protein expression of PPARG, VEGF-A, PI3K, p-PI3K, AKT, and p-AKT were detected by western blot.  ^*∗*^*P* < 0.05,  ^*∗*^ ^*∗*^*P* < 0.01, and  ^*∗*^ ^*∗*^ ^*∗*^*P* < 0.001 vs. control group; ^##^*P* < 0.01 and ^###^*P* < 0.001 vs. model group; ^$^*P* < 0.05, ^$$^*P* < 0.01, and ^$$$^*P* < 0.001*vs.* high-dose QYDP group.

To investigate the protective effect of QYDP on renal injury, we assessed the mRNA or protein expression of core genes and PI3K-AKT pathway genes by qRT-PCR and western blot analysis. The qRT-PCR results showed that the expressions of PPARG, ALB, and INS were decreased, while the expressions of TNF, VEGF-A, MAPK3, and JUN were increased in the model group compared with the control group. Moreover, the addition of QYDP significantly upregulated the expression of PPARG and ALB and downregulated the expression of VEGF-A and JUN, but had no effect on the expression of TNF, MAPK3, and INS. The addition of 740Y-P reversed the effect of high-dose QYDP on hub gene expression. The expression of PI3K and AKT have not changed in all groups (Figure 9(a)). Additionally, the western bolting showed that the protein expressions of PPARG, VEGF-A, PI3K, and AKT were consistent with the mRNA expression results. Compared with the control group, the expressions of p-PI3K and AKT were increased in the model group. QYDP administration markedly decreased p-PI3K and AKT expression in DN mice. While 740Y-P administration obviously weakened the inhibitory effect of high-dose QYDP (Figure 9(b)).

## 4. Discussion

Despite advances in the pathogenesis of DN, its high incidence and poor prognosis have not been greatly improved. Therefore, it is of great significance to explore new therapeutic drugs and targets for DN. In this study, network pharmacology was used to investigate the anti-DN mechanism of QYDP. Then, an STZ-induced DN mouse model was constructed. We evaluated the renal protective effect of QYDP on DN mice, detected the expression of inflammatory factors, and detected the effect of QYDP on the main drug targets in DN treatment by western blot and qRT-PCR assay.

In the present study, a total of 116 active compounds and 160 potential targets were collected of QYDP anti-DN. From the H-C-T network, we have found that palmitic acid, folic acid, quercetin, choline, oleic acid, and so on could potentially be the most important active compounds of QYDP. A study has shown that long-term exposure of cultured podocytes to palmitic acid significantly reduces the protein level of peroxidase, and in the late stage of DN, palmitic acid can cause podocyte damage through insufficient peroxidase response to H_2_O_2_ [17]. Modern pharmacological studies have shown that quercetin has various biological functions such as antioxidant, antiallergy, antiinflammatory, and antiapoptosis [18]. Quercetin has been reported to improve diabetic nephropathy caused by streptozotocin [19]. Moreover, astragaloside, the active component of Radix Astragali, has potent antioxidant effects *in vitro* and can inhibit the proliferation of mesangial cells induced by high glucose [20, 21]. Redroot sage extract was renoprotective in streptozotocin-induced diabetic rats [22], improved renal function, and reduced TGF-*β*1 [23] and collagen IV [24].

Moreover, from the hub genes network, we have obtained 10 targets associated with QYDP against DN, including AKT1, TNF, ALB, INS, PPARG, IL-6, IL-1B, VEGF-A, JUN, and MAPK3. The protein kinase AKT regulates a variety of cellular functions, such as glucose metabolism, cell growth, and cell survival. Changes in its expression or activity are involved in the development of diabetes and DN [25]. VEGF-A is an important regulator of angiogenesis and vascular permeability and may play a pathogenic role in DN [26]. When VEGF-A expression is below normal levels, podocytes are damaged [27]. PPARG is a ligand-activated transcription factor, and studies have found that PPARG variants contribute to the development of DN in T2DM [28]. Importantly, in our study, the qRT-PCR and western blot results suggested that QYDP could regulate the expression of hub targets, including RRARG, VEGF-A, and p-AKT.

Furthermore, the GO enrichment analysis revealed QYDP anti-DN may be associated with antioxidant activity. Studies have shown that oxidative stress plays a crucial role in DN, and excessive oxidative stress can lead to glomerulosclerosis and renal fibrosis, ultimately leading to DN [16, 29]. The KEGG enrichment analysis revealed that QYDP anti-DN may through the AGE-RAGE signaling pathway in diabetic complications, PI3K-AKT signaling pathway, TGF-*β* signaling pathway, and so on. Most targets are distributed among multiple pathways, including oxidative stress, inflammation, metabolism, the immune system, and apoptosis. Studies have shown that hyperglycemia-induced oxidative stress and inflammation are key contributors to kidney injury and nephropathy [30, 31]. Several studies have demonstrated that the AGE-RAGE signaling pathway is involved in the pathogenesis of diabetes and its complications [32]. It can exacerbate diabetes-induced vascular damage through oxidative stress [33]. The PI3K-AKT signaling pathway has been shown to be a source of glomerular hypertrophy and extracellular matrix accumulation [34]. PI3K-AKT signaling pathway plays a key role in extracellular matrix accumulation, mesangial cell proliferation, and epithelial-mesenchymal transition [35, 36]. In this study, QYDP could decrease the expression of inflammatory cytokines (IL-1*β*, IL-6, and TNF-*α*) in the serum of the DN mice and the western blotting suggested that QYDP could inhibit the PI3K-AKT signaling pathway. These results suggest that QYDP could treat DN by inhibiting the PI3K-AKT signaling pathway.

In conclusion, the network pharmacology analysis showed that QYDP has 116 active compounds and 160 targets against DN. GO enrichment analysis revealed that QYDP anti-DN may be associated with antioxidant activity. KEGG enrichment analysis found that multiple pathways involved in QYDP alleviated DN, such as the AGE-RAGE signaling pathway in diabetic complications and the PI3K-AKT signaling pathway. Animal experiments suggest that QYDP may treat DN by reducing inflammation and oxidative stress levels and inhibiting the PI3K-AKT signaling pathway. Notably, our findings partially elucidate the complex anti-DN mechanisms in QYDP and provide insights into the efficacy and drug activity of QYDP.

## Data Availability

The data used to support the findings of this study are available from the corresponding author upon request.

## Supplementary Materials

Supplementary Materials Supplementary Table 1: the primer sequences for qRT-PCR.
