# Supplementary material for: Qishen Yiqi Dripping Pill Protects Diabetic Nephropathy by Inhibiting the PI3K-AKT Signaling Pathways in Rats
Source: Evid Based Complement Alternat Med. 2022 Jun 17;2022:6239829. doi: 10.1155/2022/6239829 (PMC9232344; doi:10.1155/2022/6239829)
Supplement: Supplementary Materials — Supplementary Table 1: the primer sequences for qRT-PCR. [file 6239829.f1.docx]

Supplementary Table 1: The primer sequences for qRT-PCR

| Genes | primer sequences (5’-3’) |
| --- | --- |
| TNF | F: CAGGCGGTGCCTATGTCTC |
|  | R: CGATCACCCCGAAGTTCAGTTCAGTAG |
| INS | F: TAACCCCCAGCCCTTAGTGACCAGCTATAA |
|  | R: AAAGTTTTATTCATTGCAGAGGGGTGGGGC |
| ALB | F: TGCTTTTTCCAGGGGTGTGT |
|  | R: CATGGTGTCATGCCTCCACCT |
| TNF-α | F: CAGGCGGTGCCTATGTCTC |
|  | R: CGATCACCCCGAAGTTCAGTTCAGTAG |
| PPARG | F: GGGGATGTCTCACAATGCCA |
|  | R: GATGGCCACCTCTTTGCTCT |
| MAPK3 | F: TCCGCCATGAGAATGTTATAGGC |
|  | R: GGTGGTGTTGATAAGCAGATTGG |
| VEGF-A | F: CTGCCGTCCGATTGAGACC |
|  | R: CCCCTCCTTGTACCACTGTC |
| JUN | F: TTCCTCCAGTCCGAGAGCG |
|  | R: TGAGAAGGTCCGAGTTCTTGG |
| AKT | F: CGCTTCTATGGTGCGGAGAT |
|  | R: AACAGCTTCTCGTGGTCCTG |
| PI3K | F: CCACGGTTTGGACTATGGAA |
|  | R: ACCATTCAGCATCCTGCAAG |
| GAPDH | F: TGTGGGCATCAATGGATTTGG |
|  | R: ACACCATGTATTCCGGGTCAAT |
